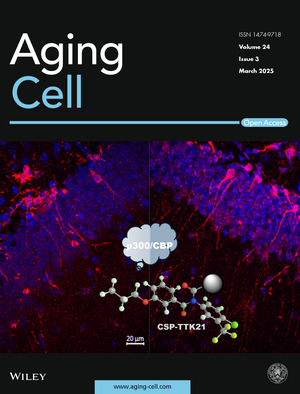# Featured Cover

**DOI:** 10.1111/acel.70045

**Published:** 2025-03-11

**Authors:** Akash Kumar Singh, Ila Joshi, Neeharika M. N. Reddy, Sushmitha S. Purushotham, M. Eswaramoorthy, Madavan Vasudevan, Sourav Banerjee, James P. Clement, Tapas K. Kundu

## Abstract

The cover image is based on the article *Epigenetic modulation rescues neurodevelopmental deficits in Syngap1+/−; mice* by Akash Kumar Singh et al.,https://doi.org/10.1111/acel.14408